# An improved cell separation technique for marine subsurface sediments: applications for high-throughput analysis using flow cytometry and cell sorting

**DOI:** 10.1111/1462-2920.12153

**Published:** 2013-06-03

**Authors:** Yuki Morono, Takeshi Terada, Jens Kallmeyer, Fumio Inagaki

**Affiliations:** 1 Geomicrobiology Group Kochi Institute for Core Sample Research, Japan Agency for Marine-Earth Science and Technology (JAMSTEC)Monobe B200, Nankoku, Kochi, 783-8502, Japan; 2 Marine Works Japan LtdOppamahigashi 3-54-1, Yokosuka, 237-0063, Japan; 3 Deutsches GeoForschungsZentrum GFZSection 4.5 Geomicrobiology, Telegrafenberg, 14473, Potsdam, Germany

## Abstract

Development of an improved technique for separating microbial cells from marine sediments and standardization of a high-throughput and discriminative cell enumeration method were conducted. We separated microbial cells from various types of marine sediment and then recovered the cells using multilayer density gradients of sodium polytungstate and/or Nycodenz, resulting in a notably higher percent recovery of cells than previous methods. The efficiency of cell extraction generally depends on the sediment depth; using the new technique we developed, more than 80% of the total cells were recovered from shallow sediment samples (down to 100 meters in depth), whereas ∼ 50% of cells were recovered from deep samples (100–365 m in depth). The separated cells could be rapidly enumerated using flow cytometry (FCM). The data were in good agreement with those obtained from manual microscopic direct counts over the range 10^4^–10^8^ cells cm^−3^. We also demonstrated that sedimentary microbial cells can be efficiently collected using a cell sorter. The combined use of our new cell separation and FCM/cell sorting techniques facilitates high-throughput and precise enumeration of microbial cells in sediments and is amenable to various types of single-cell analyses, thereby enhancing our understanding of microbial life in the largely uncharacterized deep subseafloor biosphere.

## Introduction

Obtaining a complete understanding of the nature and extent of microbial communities in the subsurface biosphere remains an ongoing challenge for microbial ecologists. The ability to detect microbes and precisely characterize microbial communities *in situ* in geological habitats is of fundamental importance in meeting this challenge; however, analyzing the microbiota in deep and ancient sedimentary niches presents significant challenges because of the extremely low metabolic activity and abundance of these organisms (D’Hondt *et al*., 2002; 2004).

New techniques for sample preparation and cell counting have been developed in recent years in order to obtain accurate and reliable enumerations of microbial cells in sedimentary habitats. These new techniques include use of gentle centrifugation (Lunau *et al*., 2008) and bilayer density separation and filtration to separate cells from sediment particles (Kallmeyer *et al*., 2008). These methods were developed for manual microscopic cell counting, and require a great deal of time and effort to produce statistically meaningful data. In addition, the efficiency of cell separation/extraction with these methods depends largely on sample quality (i.e. sediment lithology, biomass) and the skill of the analyst.

Another drawback to microscopic manual cell counting methods involves recognition of cell-derived fluorescent signals from non-biological background noise. In addressing this issue, we previously demonstrated that SYBR Green I-stained cells have a different fluorescent pattern that is clearly distinguishable from non-biological fluorescent signals produced by SYBR-stainable particulate matter (hereafter, SYBR-SPAM). Most SYBR-SPAMs produce longer wavelength signals than do SYBR-stained cells, and these longer wavelength signals can be eliminated by processing the green- and red-filtered fluorescent images (Morono *et al*., 2009).

Although recognition of cells when using an image-based cell enumeration method is independent of human variation, researchers must be extremely careful to avoid contamination during the preparation of membrane filters. Even though an automated robotic slide-loader system has been developed for acquisition of images from multiple filters (Morono and Inagaki, 2010), some technological and/or methodological improvements are needed to resolve the following issues: (i) filter preparation requires very careful aseptic handling under clean experimental conditions, and preparing filters onboard a ship is therefore rather difficult; (ii) the robotic microscopic system is lab-customized and hence not available in most microbiology laboratories and (iii) a longer operation time is needed to acquire images with the automatic z-focus adjustment. These issues become particularly critical when obtaining images of low biomass (< 10^4^ cells cm^−3^) samples.

Flow cytometry (FCM) is a powerful tool for identifying and enumerating fluorescent-labelled cells on the basis of size, fluorescence intensity and wavelength. Flow cytometry is commonly used in medical sciences and has been used to study the ecology of microbial communities in a variety of aquatic environments (Porter *et al*., 1995; Miteva and Brenchley, 2005; Wang *et al*., 2010; Irvine-Fynn *et al*., 2012). However, to date, it has been unfeasible to analyse sediment and soil samples using FCM because of analytical interference associated with non-biological particles such as mineral grains.

In this study, we developed an improved cell separation technique employing FCM by examining various marine sediments in order to optimize the cell separation efficiency. The combined use of our newly developed cell separation method and FCM/cell sorting makes it possible to conduct accurate and high-throughput cell enumeration and previously impractical single-cell analyses of the deep sedimentary biosphere.

## Results and discussion

### Standardization of the simple sieving FCM cell counting method for high-biomass samples

**Figure 1:**
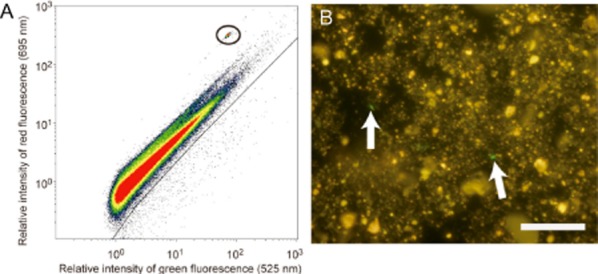
FCM cytograms (A) and microscopic observation (B) of the sediment sample stained with SYBR Green I. A volume of 0.1 × 10^−3^ cm^3^ of the sediment was stained with SYBR Green I. Signals surrounded by a solid circle are from volume calibration beads.

In general, FCM has a narrow flow path, through which small objects in the sample suspension can pass. The diameter of the narrowest path is typically around 100–200 μM. Because large (> 100 μM) mineral grains in sediment samples will get stuck in the flow path, they must be removed prior to the analysis. We attempted simple protocol, in which the sample was sieved with a 100 μm of mesh to remove large particles and then directly analysed using FCM. Figure 1A shows a scatter plot of the fluorescence intensity (cytogram) of a shallow subseafloor sample [Site C9001 Hole C, Core 3H-1, 17.7 m below seafloor (mbsf)] that was sieved and stained with SYBR Green I. By plotting the green (525 nm) and red (695 nm) fluorescence signals for particles, we were able to distinguish signals produced by microbial cells (which have greener fluorescence) from signals produced by SYBR-SPAMs (Fig. 1B). When we stained a small amount of sediment (less than 0.5 × 10^−3^ cm^3^), the signals from microbial cells were distinguishable from those of SYBR-SPAMs, and the FCM counts were highly consistent with those obtained using image-based microscopic counting (Table 1). However, when we increased the amount of sediment for staining, the FCM cell counts decreased markedly, simultaneously, the signal from SYBR-SPAMs became greener, changing the signal pattern accordingly (Fig. S1B–G). Using a microscope, we carefully examined the sediment suspension after FCM analysis and found that a certain fraction of the cells were not stained; Fig. S2A–G shows the results of microscopic observation of membranes on which varying amounts of sediment were stained in suspension. When more than 2.0 × 10^−3^ cm^3^ of sediment was applied, no clearly distinct green cells were observed in the microscopic image, and the colour of the SYBR-SPAMs gradually turned to be green (Fig. S2D–G). However, after restaining the membranes shown in Fig. S3, we observed clear green-fluorescing microbial cells and red-shifted fluorescence of SYBR-SPAMs. This result demonstrates three things: (i) the amount of SYBR Green I dye was not enough to obtain clear discrimination of microbial cells and SYBR-SPAMs, (ii) non-specific adsorption of the SYBR Green I dye to SYBR-SPAMs prevailed against the formation of the SYBR Green I-DNA complex within the cell and (iii) the red shift in the fluorescence spectrum of SYBR Green I on SYBR-SPAM is the result of high accumulation of the dye (Morono *et al*., 2009). When non-biological grains adsorb a significant amount of SYBR Green I dye, the effective concentration of free dye molecules decreases, resulting in failure or low efficiency of intracellular DNA staining. A similar trend was observed on membranes stained with low concentrations of SYBR Green I (Fig. S3). Therefore, optimization of the ratio between dye concentration and sample amount is critical when counting sedimentary microbial cells.

**Table 1:** Cells count as determined from FCM analysis of various amounts of sediment stained with SYBR Green I after simple sieving. Data are shown as the mean ± standard deviation of four replicates

Sediment amount applied for staining (× 10^−3^ cm^3^)	Cell count (× 10^8^ cells cm^−3^)
0.1	2.01 ± 0.19
0.2	2.27 ± 0.14
0.5	2.16 ± 0.10
1	0.67 ± 0.07
2	0.34 ± 0.07
5	0.14 ± 0.01
10	0.02 ± 0.01
Membrane-based count	2.24

From a practical standpoint, our protocol allows staining of up to 0.2 × 10^−3^ cm^3^ of sediment to give efficient and specific cell counting with FCM within a reasonable analysis time (10–20 min) per sample for high-throughput cell enumeration. Assuming that ∼ 100 cells must be counted per analysis to provide satisfactory statistical confidence, the amount of sediment noted above would correspond to over 5.0 × 10^5^ cells cm^−3^. The simple sieving protocol described in the present report (see *Experimental procedures*) can be employed only for the analysis of relatively high-biomass sediments (e.g. shallow sedimentary habitats). The use of FCM significantly increases the speed of cell counting relative to previous microscopy-based approaches.

### Bilayer cell extraction and its efficiency

To evaluate microbial populations in low-biomass sediment samples, we have tried to separate cells from sediment grains using bilayer density-based techniques. By employing a density cushion beneath the sample slurry, microbial cells, which are less dense than sediment particles, can be trapped along the density boundary (i.e. just above the high-density solution) upon centrifugation. In this study, we evaluated the efficiency of the bilayer cell extraction protocol described by Kallmeyer and colleagues (2008).

**Figure 2:**
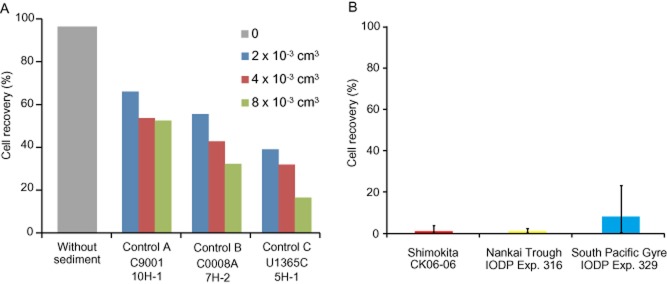
Verification of cell recovery rate using the bilayer cell separation method.A. Percent recovery of cells from control sediment A (Site C9001, Core 10H-1), B (Site C0008A, Core 7H-2) and C (Site U1365C, core 5H-1) mixed with cultured *Escherichia coli* cells. Varying amounts (2, 4, 8 × 10^−3^ cm^3^) of sediment were utilized in the separation.B. Percent recovery of cells from natural sediment samples. The number of microbial cells in 10 samples from CK06-06, 5 samples from IODP Expedition 316 and 10 samples from IODP Expedition 329 was determined without and with bilayer separation, and the average percent recovery for each sample location is shown. Bars show the maximum and minimum percentage of recovery.

To rule out the possibility that cells would adhere to and co-precipitate with sediment grains, we first evaluated mixtures of cultured and fixed *Escherichia coli* cells and cell-free sediments treated with sodium hypochlorite to remove indigenous cells (model sediment samples A, B, C; see *Experimental procedures*). The percentage of cell recovery ranged from 20% to 60% (Fig. 2A), depending on the type and amount of sediment examined. Though the recovery varied, we consistently observed a trend in which analysis of larger amounts of sediment resulted in a lower percentage of cells recovered. We also confirmed that the fraction of cells that was lost remained in the layer containing the precipitated sediment (data not shown). In contrast, the recovery in control extraction experiments using *E. coli* cells without sediment was high, around 95%. These results demonstrate that sediment particles have an effect on co-precipitation of *E. coli* cells, even across the density layer interface. Although it is possible that cells may be pushed into the heavy density layer by sediment particles, it is most likely that cells are captured in the turbulent flow behind the sediment particles as they cross the density interface, thereby drawing cells into the higher density solution. The occurrence of this phenomenon was supported by microscopic observations, which showed that *E. coli* cells co-precipitated with sediments did not attach or adhere to the surface of the sediment particles (data not shown). When we applied this bilayer separation method to natural samples (e.g. sediment cores), we obtained a lower percent recovery than expected, ranging from < 1% to 24% (Fig. 2B), although we did not see any failure associated with attachment of cells to heavy sediment particles. After storing the formaldehyde-fixed sediment slurries for several months, in some cases, we observed a lower separation yield despite identical experimental parameters, potentially due to a density increase in cells. There appears to be no systematic relationship between age and density increase. Therefore, these results strongly underscore the necessity of improving cell separation methods.

### An improved cell extraction method using multiple density layers for low-biomass samples

To increase the efficiency of cell recovery from sediment samples, we modified the density separation procedure by addressing two critical issues impacting the recovery efficiency of the bilayer cell extraction method: (i) co-precipitation of microbial cells with sediment particles and (ii) precipitation of microbial cells with higher density. Co-precipitation of cells with sediment particles apparently occurs at the surface of high-density solutions as a result of hydrodynamic dragging of cells in the turbulent flow behind the sediment particles. A number of micro-organisms tightly associate with minerals in nature (Inagaki *et al*., 2003; Bazylinski and Frankel, 2004; Edwards *et al*., 2005; Kolinko *et al*., 2008). If deeply buried microbial cells are more dense than normal vegetative cells (e.g. *E. coli*), this could also lead to co-precipitation with sediment particles during bilayer extraction, thereby reducing the efficiency of cell recovery.

**Figure 3:**
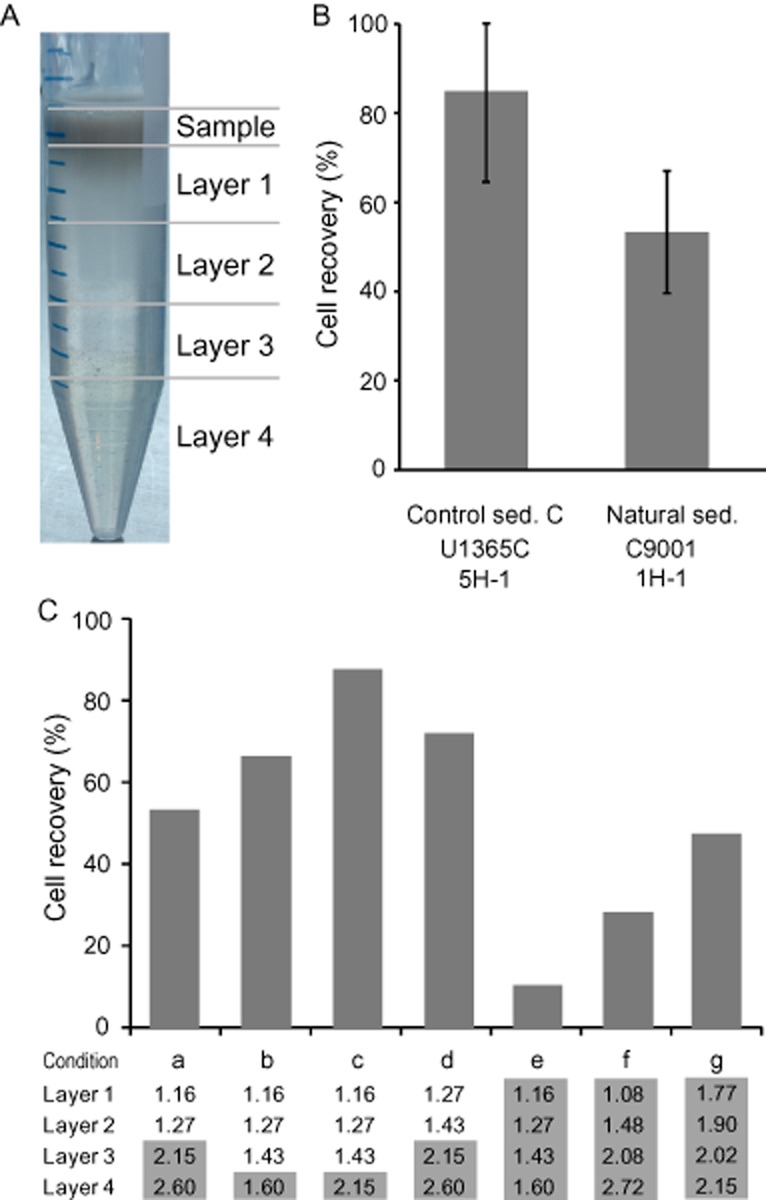
Standardization of the multilayer density separation method.A. Photograph illustrating the appearance of the multiple density layers after centrifugation.B. Percent recovery of *Escherichia coli* cells from control sediment C and natural cells from sediment sample (Site C9001, Core 1H-1). Density layers: 1.16 and 1.27 g cm^−3^ with Nycodenz and 2.15 and 2.60 g cm^−3^ with sodium polytungstate. Bars show the standard deviation (n > 10).C. Optimizing the number of density layers. Seven different composition of density layers (a–g) were compared for cell separation efficiency. The density (g cm^−3^) of each layer is shown below the bar graph. Values shaded in gray represent layers of sodium polytungstate.

We examined the use of multiple density layers of one or more relatively high-density solutions as a means of preventing the loss of cells through co-precipitation during cell separation (Fig. 3A). Sodium polytungstate, which has a maximum density of 3.1 g cm^−3^, was used to prepare a solution with a higher density than Nycodenz, which has a maximum density of 1.43 g cm^−3^ at 80% (w/v). We first evaluated the separation of *E. coli* cells using control sediment C, for which we obtained the lowest recovery with the bilayer separation method (Fig. 2A). Using Nycodenz density layers of 1.16 and 1.27 g cm^−3^ and sodium polytungstate layers of 2.15 and 2.60 g cm^−3^, the recovery of *E. coli* cells from control sediment C was 84%, which was 2.6 times higher than that obtained using bilayer separation (Fig. 3B). This result demonstrated that the use of multiple density layers is more effective than the use of a simple bilayer separation. The percent recovery of cells from natural sediment was 54%, which was 5.3 times higher than that obtained using simple bilayer separation. Despite this improvement, roughly a half of the total number of cells was lost, possibly having been retained in the high-density solution.

To improve the method further, we optimized the composition of the density layers. When we used multiple Nycodenz and sodium polytungstate layers of the same density (Fig. 3C, conditions b and e), we found that recovery was higher with Nycodenz. Theoretically, turbulent flow occurs when the Reynolds value is high. Because the viscosity of sodium polytungstate is lower than that of Nycodenz, the Reynolds value for a sodium polytungstate solution is relatively higher than that for a Nycodenz solution. By assuming that the hydrodynamics around moving particles in a sodium polytungstate solution would be dominated by turbulent flow to a greater degree than in a Nycodenz solution of the same density, we could explain the observed difference in cell recovery obtained when using two different solutions to prepare layers of the same density.

Interestingly, we achieved good cell recovery using a density combination including a layer of high density (i.e. 2.15 g cm^−3^) in which some floating sediment particles were found. In bilayer separations or multilayer separations involving low-density solutions, we often observed tightly compacted sediment appearing as a precipitate at the bottom of the tube (Fig. S4A). In contrast, by increasing the density of the lowermost layer to 2.15 g cm^−3^, a portion of the sediment particles floated at the layer interface and the amount of precipitate at the bottom of the tube was reduced (Fig. S4B). The cells, which were carried into the high-density layers along with the sediment particles, would not be able to float upward into the lower density layers because of the presence of the compacted precipitate. Therefore, having loosely packed sediment through the use of high-density layers should refocus the cells to their correct density layer, resulting in higher recovery.

**Figure 4:**
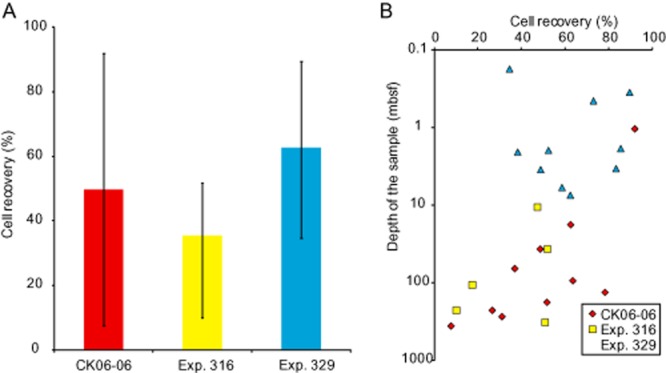
Percent recovery of cells from natural sediment samples using the multilayer density separation technique.A. Percent recovery of cells from the sediment samples described in Fig. 2B. Bars show the maximum and minimum percentage of recovery.B. Depth profile of cell recovery.

Next, we examined recovery of cells from natural sediment samples using the optimized protocol. The percent recovery of cells from all samples tested was substantially greater than that obtained with the bilayer cell separation method (Fig. 4A, c.f., Fig. 2B). The average recovery of cells from the organic-rich hemipelagic sediments collected off the Shimokita Peninsula, the silty clay from the Nankai Trough subduction zone and the organic-poor metalliferous sediments from the South Pacific Gyre was 49.8%, 35.4% and 62.7% respectively. The percent recovery from these very different samples from the continental margin and open Pacific were 45.6, 32.1 and 7.8 times higher, respectively, than those obtained using the bilayer protocol. Although we generally recovered a relatively high percentage of cells from shallow sediments, the percent recovery of cells from deep sediments was relatively low or variable (Fig. 4B). For example, the average percent recovery from shallow (0–100 mbsf) sediment samples was 60.5%, whereas the average percent recovery from deep (100–359 mbsf) sediment samples was 39.5%. Only 7.5% of cells were recovered from the deepest sample we evaluated in this study [Site C9001, Core 40H-3, 359 mbsf (Table S1)]. The low recovery of cells was presumably due to the high density of cells in the deeper subseafloor. Using higher density solutions for separation, we may expect to recover a higher percentage of these cells; however, cell separation from light sediment particles remains challenging.

### Counting and sorting cells in density-separated cell suspensions

**Figure 5:**
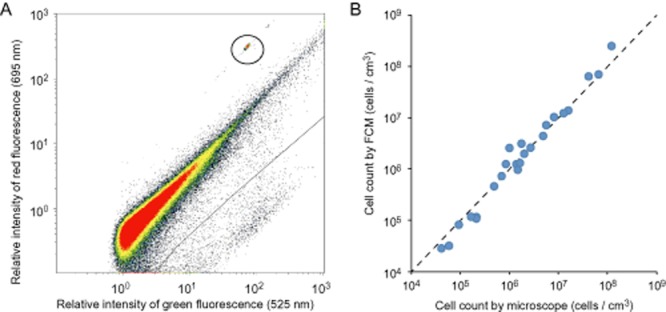
FCM analysis of density-separated sediment samples.A. FCM cytogram (scatter plot) of the sample from Site C9001, Core 1H-1 following separation and staining with SYBR Green I. Lower right portion below the solid line shows the region of cell-derived fluorescence signals. Signals surrounded by a solid circle are from volume calibration beads.B. Number of microbial cells as determined by microscopic counting and FCM. Dotted line shows the 1:1 line for the counts determined by microscopy and FCM.

Using FCM, we counted microbial cells in density-separated suspensions. Among the parameters that could be plotted on the X- and Y-axes of fluorescence intensity plots, we concluded that plotting the relative intensity of green (525 nm) versus red (625 nm) fluorescence was the best way to selectively define the region of fluorescent signals derived from cells and SYBR-SPAMs (Fig. 5A). The results of FCM cell counting analyses were in good agreement with those of manual and image-based microscopic counting over the range 10^4^–10^8^ cells cm^−3^ (Fig. 5B). An entire 500 μL of cell suspension could be processed in approximately 1000 s (∼17 min) using the Gallios flow cytometer (Beckman Coulter, Brea, CA, USA). We performed intensive cleaning steps between each FCM analysis (i.e. washing with sodium hypochlorite solution and water for 4 min) to prevent cross-contamination between samples. The relatively short analysis time of FCM compared with manual microscopic counting (up to several hours per sample) demonstrates that FCM is a valuable tool for high-throughput counting of microbes in samples from sedimentary environments.

**Figure 6:**
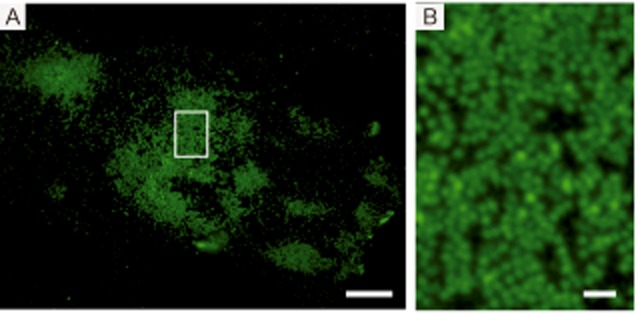
Density-separated and selectively sorted cells from South Pacific Gyre (Site U1365, Core 1H-1, 0.45 mbsf) sediment. More than 10^5^ microbial cells were sorted and accumulated within a very small area of the membrane filter. Bar: 20 μm (A), 2 μm (B).

The ability to discriminate between microbial cells using the density-based cell separation technique can also be applied to the isolation and purification of individual cells by incorporating the instrument with cell sorting function (Moflo, Beckman Coulter). Using the same fluorescence conditions used in the FCM analyses, we selectively sorted more than 10^5^ microbial cells from South Pacific Gyre sediment (Site U1365, Core 1H-1, 0.45 mbsf), in which the cell abundance was ∼ 10^5^ cells cm^−3^ (D’Hondt *et al*., 2009; Kallmeyer *et al*., 2012) (Fig. 6). Because of the low number of cells in the sample we examined, several hours were required to sort ∼ 10^5^ cells; however, less time would be required when sorting sediment samples with higher concentrations of cells. It is also worth noting that as the fraction of SYBR-SPAMs in a sample increases, the efficiency of cell sorting (i.e. a number of sorted cells per second) decreases. Therefore, to keep the high efficiency of cell sorting, separating microbial cells are advantageous over cell sorting with non-separated sediments.

## Conclusions and future applications

In this study, we developed an improved technique for separating microbial cells from marine subsurface sediments and standardized methods for discriminative and high-throughput cell enumeration using FCM. The ability to differentiate SYBR-stained cells from SYBR-SPAMs on FCM cytograms ensures repeatable, objective, rapidly obtained and reliable data that do not depend on manual visual observation. Especially for the analysis of low-biomass samples such as sediments from deep and/or ultra-oligotrophic environments, the multilayered density separation technique described here provides a much improved efficiency of cell recovery.

The application of selective cell recognition in FCM and cell sorter for sorting target cells will open a new window to (but not limited to) the largely uncharacterized subseafloor and deep biosphere at both the single cell and community levels. For example, individually sorted sedimentary microbial cells can be subjected to other powerful and high-resolution analytical approaches, such as NanoSIMS (Morono *et al*., 2011), single cell genomics (Stepanauskas, 2012) and/or cell-level elemental analyses (Eek *et al*., 2007).

## Experimental procedures

### Sediment core sampling

Marine subsurface sediment core samples were collected on the D/V *Chikyu* Shakedown Cruise CK06-06 off the Shimokita Peninsula of Japan in 2006 (Site C9001 Hole C) (Aoike, 2007; Masui *et al*., 2008; Morono *et al*., 2009; 2011), from the Nankai Trough plate subduction zone during Integrated Ocean Drilling Program (IODP) Expedition 316 in 2008 (Site C0006 Hole A) (Kimura *et al*., 2008) and from the South Pacific Gyre during IODP Expedition 329 in 2010 (Site U1365 Hole C) (Expedition 329 Scientists, 2011). The samples from CK06-06 and IODP Expedition 316 were taken onboard and immediately fixed with 2% paraformaldehyde for about 6 h at 4°C. The fixed slurry samples were washed twice with phosphate buffered saline (PBS), resuspended in 10 ml of PBS-ethanol (1:1) solution and stored at −20°C. Prior to laboratory use, the slurry samples were centrifuged at 4500 × *g* for 15 min, after which the supernatant was discarded and the pellet resuspended by adding an equal volume of 2.5% NaCl solution. The samples from IODP Expedition 329 were taken onboard and immediately fixed with 2% formaldehyde, stored at 4°C and used directly without washing.

For optimization of the cell separation protocol, we prepared control sediment samples by mixing a known number of *E. coli* cells with ‘cell/DNA-free’ control sediments. Control sediments were prepared by treating frozen samples of sediment core 10H-1 from Site C9001 Hole C (84.0 mbsf, control sediment A), Core 7H-2 from Site C0008 hole A (54.9 mbsf, control sediment B) and Core 5H-1 from Site U1365 hole C (35.5 mbsf, control sediment C) with an equal volume of a commercial HClO-based cleaning product overnight, after which the samples were washed five times with TE buffer [10 mM Tris-HCl, 1.0 mM ethylenediaminetetraacetic acid (EDTA), pH 8.0] containing 0.1% (w/v) Na_2_S to ensure that no unreacted hypochloride remains in the sample.

### Cell detachment and density-based cell separation

Sediment slurry (250 μl) was diluted with 150 μl of 2.5% NaCl solution, and 50 μl of detergent mix [100 mM EDTA, 100 mM sodium pyrophosphate, 1% (v/v) Tween 80] and 50 μl methanol were added. Next, the sample was vigorously shaken for 60 min at 500 r.p.m. using a Shake Master (Bio Medical Science, Tokyo, Japan). After shaking, the sediment slurry was sonicated at 20 W for 1 min using a Model UH-50 Ultrasonic Homogenizer (SMT, Tokyo, Japan), and then carefully layered onto a high-density cushion solution. Either 50% (w/v) Nycodenz (Kallmeyer *et al*., 2008) or various combinations of 50–80% (w/v) Nycodenz and 40–80% (w/v) sodium polytungstate were used to prepare the high-density solutions. Samples were centrifuged at either 4500× *g* or 15 000× *g* for 15–300 min, after which the supernatant, including the high-density layer(s), was carefully removed and transferred to a separate vial. Next, 900 μl of 2.5% NaCl solution was added to the remaining high-density solution and sediment pellet, which was resuspended and centrifuged again at 5000× *g* for 15 min. The resulting supernatant was also transferred to a separate vial. The remaining pellet was resuspended in 100 μl of 1% hydrofluoric acid and allowed to stand for 20 min. The reaction was stopped by adding 100 μl of 1.5 M Tris-base, and the sample was shaken again for 10 min after addition of 150 μl of 2.5% NaCl solution and 50 μl each of detergent mix and methanol. The vial was then sonicated in a water bath for 30 s, and layering onto the high-density solution and subsequent centrifugation steps were repeated as described above.

### Membrane-based cell counting

The supernatant from the density centrifugation step was filtered using a 0.22 μm of pore size black polycarbonate membrane (EMD Millipore, Billerica, MA, USA). About 5 ml of filtered (0.22 μm) 2.5% NaCl solution was placed into the filter tower prior to the addition of the supernatant to ensure an even distribution of cells on the filter. The membrane was then washed with 5 ml of TE buffer and roughly 2 × 10^8^ fluorescent microsphere beads [Fluoresbrite Bright Blue Carboxylate Microspheres (BB beads), 0.5 μm, Polysciences, PA, USA] were added for use in focus adjustment (Morono *et al*., 2009). After air-drying, a quarter of the membrane was placed on the filtration device again and stained with SYBR Green I [1/40 (v/v) SYBR Green I in TE buffer]. The stained filter was finally mounted on a glass microscope slide with 3–5 μl of mounting solution [2:1 mixture of VECTASHIELD mounting medium H-1000 (Vector Laboratories, Burlingame, CA, USA) and TE buffer]. Microscopic fluorescence image acquisition [at 525/36 nm (center wavelength/bandwidth) and 605/52 nm × 490 nm excitation] was performed automatically using a fluorescence microscope system equipped with an automatic slide handler (Morono and Inagaki, 2010). The resulting images were analysed using the macro of Metamorph software (Molecular Devices, Sunnyvale, CA, USA) to discriminatively enumerate microbial cells on the membrane.

### FCM analysis and cell sorting

Microbes in deep sedimentary habitats are smaller in size than those in shallow habitats (Morono *et al*., 2009; Hinrichs and Inagaki, 2012; Kallmeyer *et al*., 2012), rendering it difficult to clearly distinguish cell-derived fluorescent signals in samples from the deep sedimentary environment. In this study, we used a Gallios high-spec flow cytometer (Beckman Coulter) to analyse the small cells found in deep sediment samples. Using FCM, we could detect 0.2 μm of fluorescent microspheres and count up to 97% of the applied microspheres (Fig. S5).

For the simple sieving protocol, 0.1–10 × 10^−3^ cm^3^ of a cell-detached sediment suspension (CK06-06 Core 3H-1) in 500 μl of TE buffer was sieved through 40 μm of mesh (Cell Strainer; BD Biosciences, Franklin Lakes, NJ, USA). Either the sieved sediment suspension or supernatants from the density centrifugation was placed onto an Anopore Inorganic Membrane (Anodisc, Whatmann, Kent, UK), washed with TE buffer and then stained with 100 μl of SYBR Green I staining solution. After staining for 5 min, the SYBR-stained cells were washed with 2 ml of TE buffer, and then the membrane was placed into a 15 ml centrifuge tube containing 2 ml of TE buffer. Cells were detached from the membrane by sonication at 20 W for 10–30 s using a Model UH-50 Ultrasonic Homogenizer (SMT). The suspension was then transferred to a new 15 ml centrifuge tube. The membrane was again washed with 1 ml of TE buffer, and the suspension was collected in the same centrifuge tube. The tube was centrifuged at 6000× *g* for 10 min, and then 2 ml of the supernatant was removed to reduce the sample volume. Loss of cells during this procedure was checked by processing samples with known cell concentrations and found to be less than 5%. For volumetric calibration, custom-made fluorescent beads [Green (505/515 nm) and Deep Red (633/660 nm) double colour] were added at a concentration of 4.5 × 10^4^ beads cm^−3^. The cell suspension was analysed using a Gallios flow cytometer (Beckman Coulter), and the FCM data were analysed using Kaluza analysis software (Beckman Coulter). Sorting of the cells was done with Moflo XDP high-speed cell sorter (Beckman Coulter).

## Supporting Information

Fig S1 FCM cytograms of the various amount of sediment sample stained with SYBR Green I. Each 0.1 (A), 0.2 (B), 0.5 (C), 1 (D), 2 (E), 5 (F) and 10 (G) (×10^-3^) cm^3^ of the sediment was stained with SYBR Green I and applied to FCM analysis. Lower right part below the solid line shows the region of cell-derived signals. The signals in a solid circle are derived from volume calibration beads.

Fig S2 Microscopic observation of the sediment used for the FCM analysis as shown in Figure S1. Each 0.1 (A), 0.2 (B), 0.5 (C), 1 (D), 2 (E), 5 (F) and 10 (G) (×10^-3^) cm^3^ of the sediment was stained with SYBR Green I. White arrows show microbial cells with green fluorescence. Bar: 20 μM.

Fig S3 Staining sediment samples with various concentration of SYBR I. Sediment sample of CK06-06, 3H-1 was stained with (A) 250×, (B) 100×, (C) 50×, (D) 20×, (E) 10×, (F) 5×, (G) 2× and (H) 1× of SYBR I solution (concentration was shown as relative to instructed standard concentration by manufacturer).

Fig S4 Differences on the floating particles after density separations depending on the heaviest density solution.

Fig S5 Analysis of 0.2 μm fluorescent microspheres with Gallios flow cytometer.

Table S1 Cell abundance and cell recoveries of natural sediment samples with conventional bilayer and improved multilayer separation methods.
