# Supplementary figures and images for: An improved cell separation technique for marine subsurface sediments: applications for high-throughput analysis using flow cytometry and cell sorting
Source: Environ Microbiol. 2013 Jun 3;15(10):2841–9. doi: 10.1111/1462-2920.12153 (PMC3910163; doi:10.1111/1462-2920.12153)

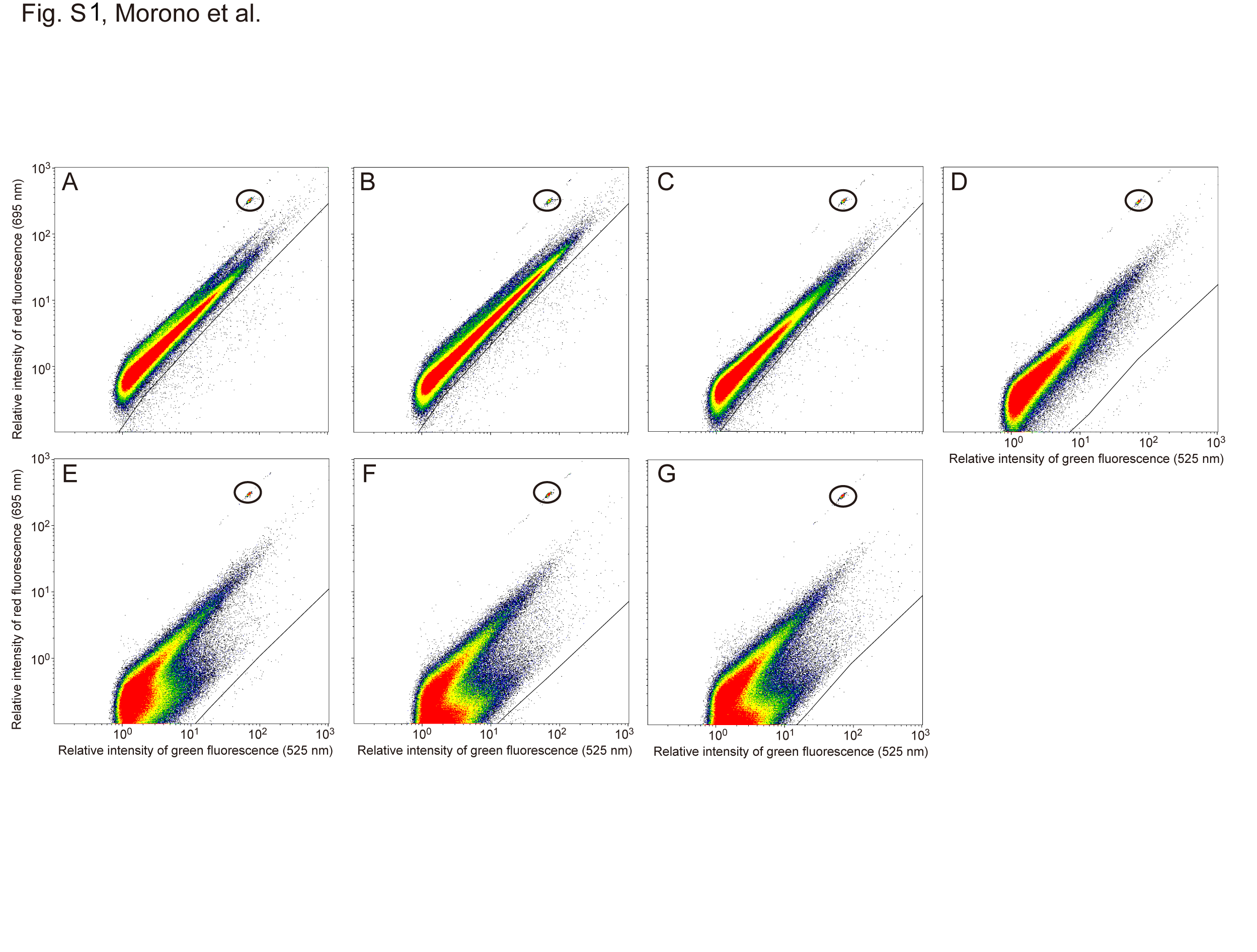

Supplement: Fig S1 — FCM cytograms of the various amount of sediment sample stained with SYBR Green I. Each 0.1 (A), 0.2 (B), 0.5 (C), 1 (D), 2 (E), 5 (F) and 10 (G) (×10-3) cm3 of the sediment was stained with SYBR Green I and applied to FCM analysis. Lower right part below the solid line shows the region of cell-derived signals. The signals in a solid circle are derived from volume calibration beads. [file emi0015-2841-sd1.tif]

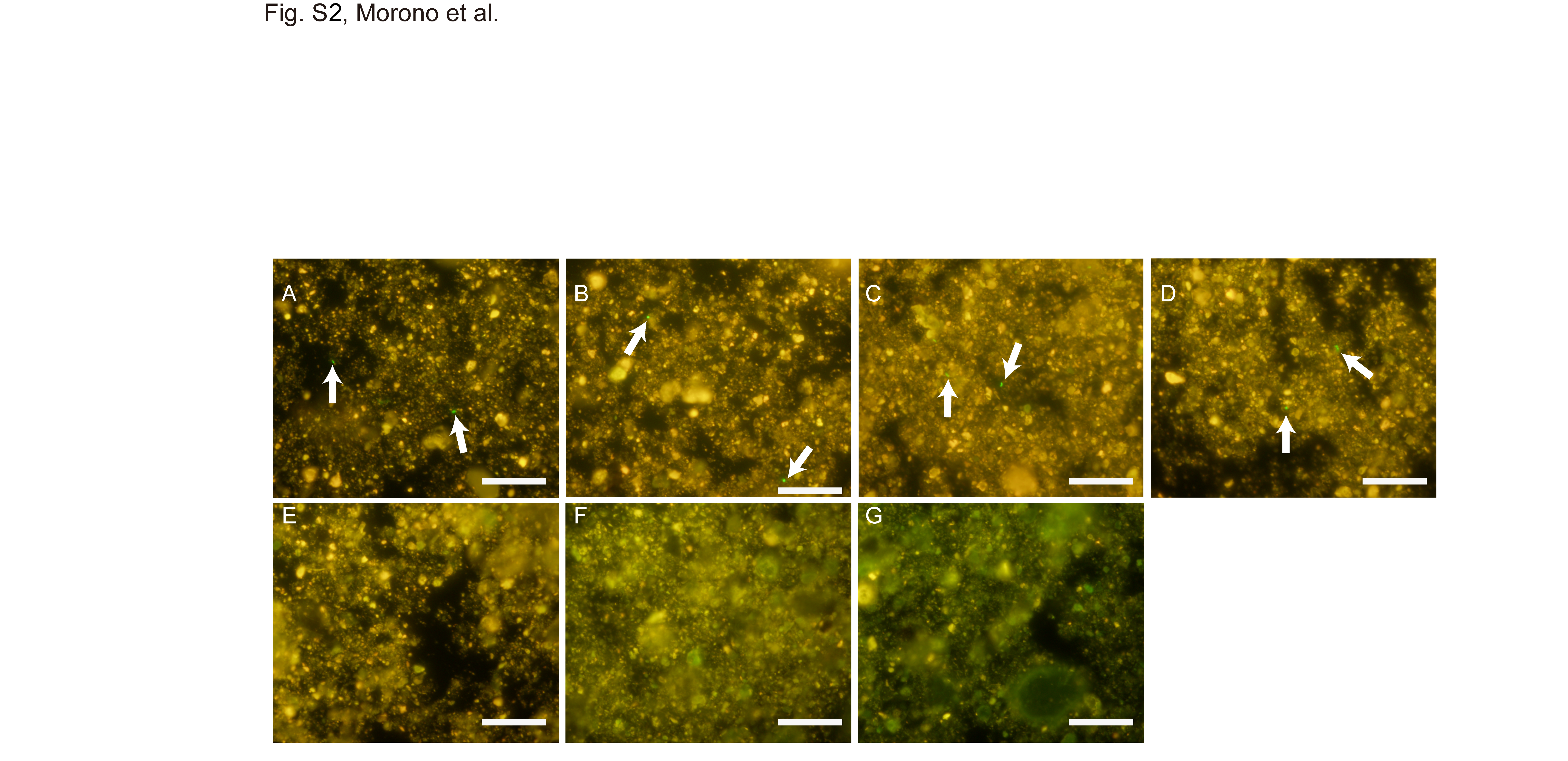

Supplement: Fig S2 — Microscopic observation of the sediment used for the FCM analysis as shown in Figure S1. Each 0.1 (A), 0.2 (B), 0.5 (C), 1 (D), 2 (E), 5 (F) and 10 (G) (×10-3) cm3 of the sediment was stained with SYBR Green I. White arrows show microbial cells with green fluorescence. Bar: 20 μM. [file emi0015-2841-sd2.tif]

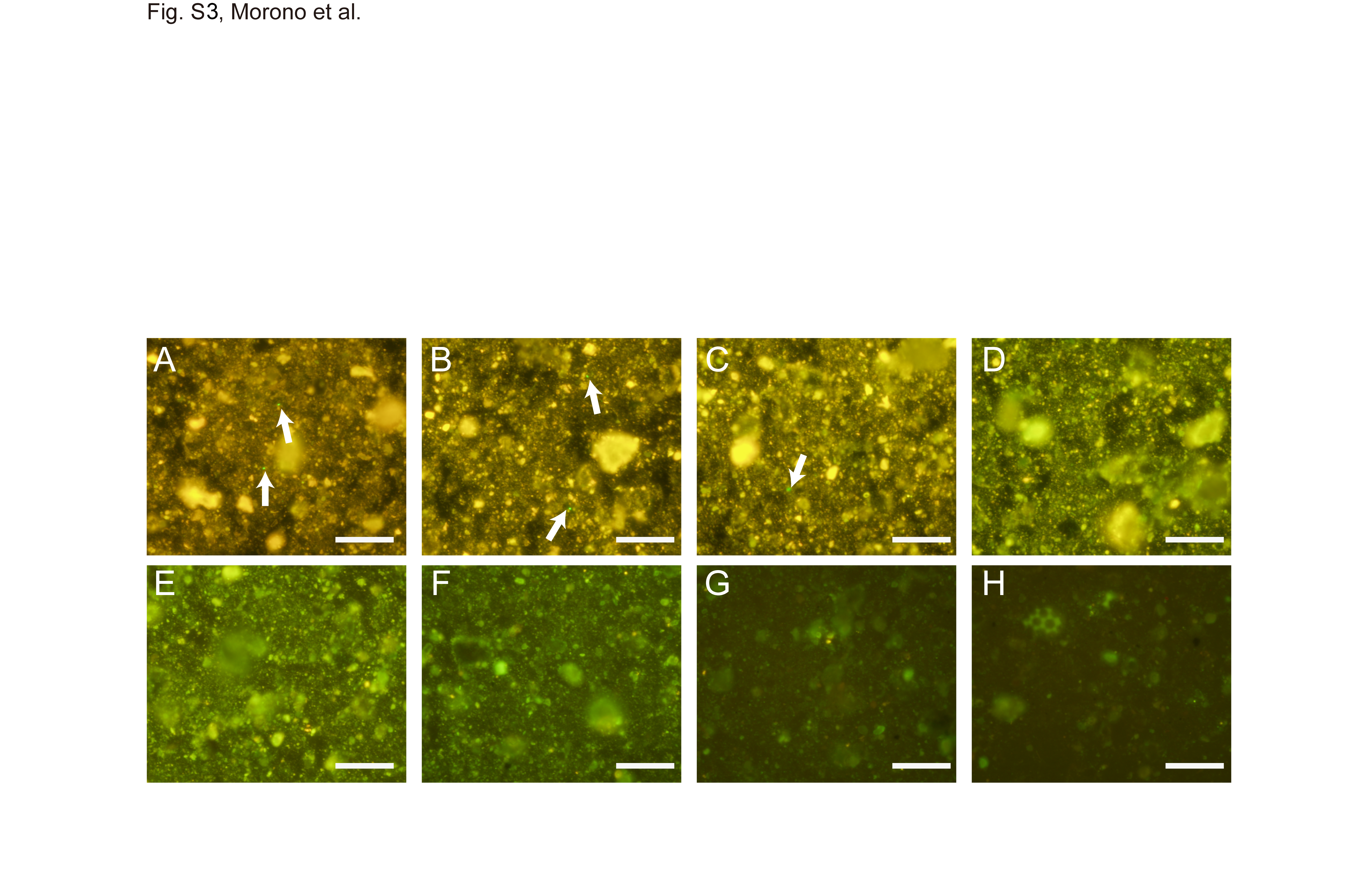

Supplement: Fig S3 — Staining sediment samples with various concentration of SYBR I. Sediment sample of CK06-06, 3H-1 was stained with (A) 250×, (B) 100×, (C) 50×, (D) 20×, (E) 10×, (F) 5×, (G) 2× and (H) 1× of SYBR I solution (concentration was shown as relative to instructed standard concentration by manufacturer). [file emi0015-2841-sd3.tif]

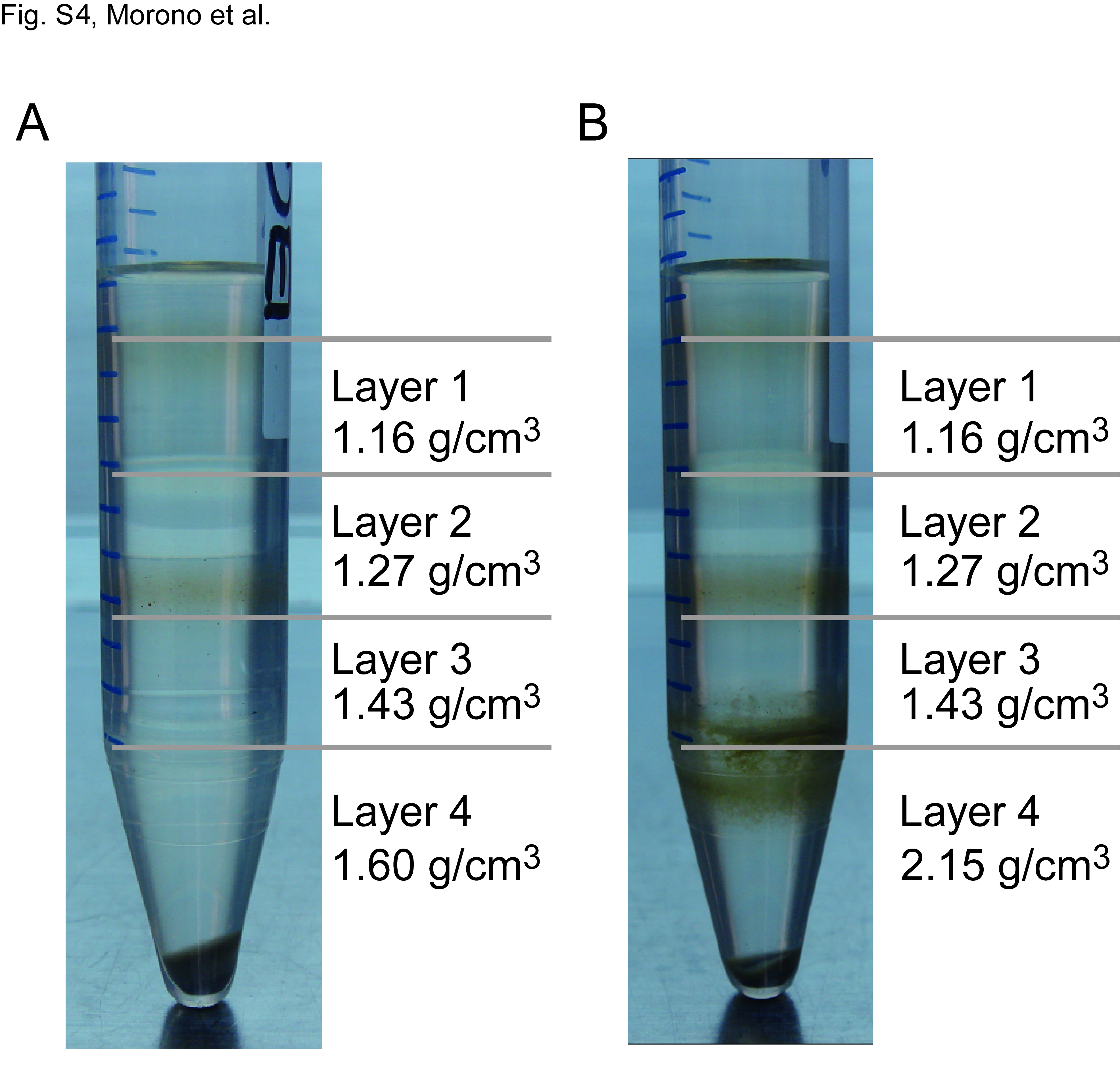

Supplement: Fig S4 — Differences on the floating particles after density separations depending on the heaviest density solution. [file emi0015-2841-sd4.tif]

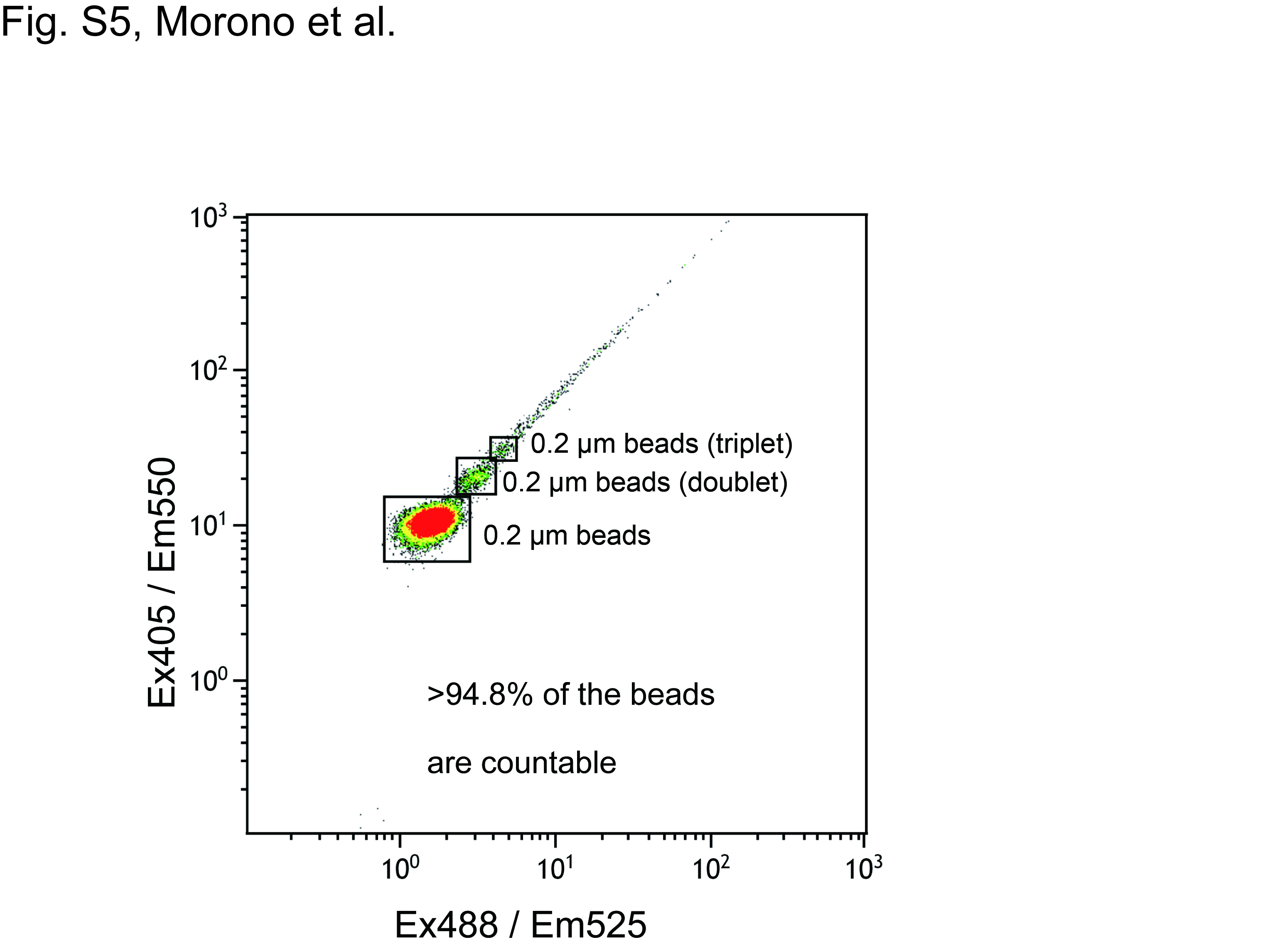

Supplement: Fig S5 — Analysis of 0.2 μm fluorescent microspheres with Gallios flow cytometer. [file emi0015-2841-sd5.tif]
